# Diaphragm ultrasound as a new index of discontinuation from mechanical ventilation

**DOI:** 10.1186/2036-7902-6-8

**Published:** 2014-06-07

**Authors:** Giovanni Ferrari, Giovanna De Filippi, Fabrizio Elia, Francesco Panero, Giovanni Volpicelli, Franco Aprà

**Affiliations:** 1 High Dependency Unit, San Giovanni Bosco Hospital, P.za Donatore del Sangue 3, Turin 10154, Italy; 2 Department of Emergency Medicine, San Luigi Gonzaga University Hospital, Turin 10043, Italy

**Keywords:** Diaphragm, Ultrasonography, Weaning

## Abstract

**Background:**

Predictive indexes of weaning from mechanical ventilation are often inaccurate. Among the many indexes used in clinical practice, the rapid shallow breathing index is one of the most accurate. We evaluated a new weaning index consisting in the diaphragm thickening fraction (DTF) assessed by ultrasound.

**Methods:**

Forty-six patients were prospectively enrolled. All patients were ventilated in pressure support through a tracheostomy tube. Patients underwent a spontaneous breathing trial (SBT) when they met all the following criteria: FiO_2_ < 0.5, PEEP ≤5 cmH_2_O, PaO_2_/FiO_2_ > 200, respiratory rate <30 breaths per minute, absence of fever, alert and cooperative, and hemodynamic stability without vaso-active therapy support. During the trial, the right hemi-diaphragm was visualized in the zone of apposition using a 10-MHz linear ultrasound probe. The patient was then instructed to perform breathing to total lung capacity (TLC) and then exhaling to residual volume (RV). Diaphragm thickness was recorded at TLC and RV, and the DTF was calculated as percentage from the following formula: Thickness at end inspiration - Thickness at end expiration / Thickness at end expiration. Also, the rapid shallow breathing index (RSBI) was calculated. Weaning failure was defined as the inability to maintain spontaneous breathing for at least 48 h, without any form of ventilatory support.

**Results:**

A significant difference between diaphragm thickness at TLC and RV was observed both in patients who succeeded SBT and patients who failed. DTF was significantly different between patients who failed and patients who succeeded SBT. A cutoff value of a DTF >36% was associated with a successful SBT with a sensitivity of 0.82, a specificity of 0.88, a positive predictive value (PPV) of 0.92, and a negative predictive value (NPV) of 0.75. By comparison, RSBI <105 had a sensitivity of 0.93, a specificity of 0.88, a PPV of 0.93, and a NPV of 0.88 for determining SBT success.

**Conclusions:**

This study shows that in our cohort of patients, the assessment of DTF by diaphragm ultrasound may perform similarly to other weaning indexes. If validated by other studies, this method may be used in clinical practice.

## Background

Difficulties in weaning from mechanical ventilation are encountered in approximately 20% of patients, and more than 40% of the time passed in the intensive care unit is spent to try to wean off from mechanical ventilation [1]. Several indexes have been employed to assess the patient's ability to recover spontaneous breathing. Variables such as minute ventilation (Ve), maximum inspiratory pressure (PImax), breathing frequency, rapid shallow breathing index (RSBI, i.e., respiratory frequency/tidal volume), tracheal airway occlusion pressure 0.1 s (P 0.1), and a combined index named CROP (compliance, rate, O_2_, pressure index) have been used in common clinical practice [2]. RSBI was found to be one of the most accurate predictors of failure [3]. However, values of sensitivity and specificity and negative predictive values for the suggested threshold of rapid shallow breathing index <105 were highly variable in different studies [4-6].

The diaphragm is a fundamental respiratory muscle whose dysfunction may be very common in patients undergoing mechanical ventilation. Diaphragm dysfunction is associated to prolonged mechanical ventilation and weaning failure [7-10]. The diagnostic tools traditionally used to study diaphragm dysfunction, like fluoroscopy, phrenic nerve conduction study, and trans-diaphragmatic pressure measurement, present some limitations and disadvantages, including the use of ionizing radiation, low availability, invasiveness, and the need for patient transportation and skilled or specifically trained operators. Recently, ultrasound has been used to evaluate diaphragmatic function [10]. Advantages of ultrasound include safety, avoidance of radiation hazards, and availability at the bedside.

Ultrasound may be used to measure diaphragmatic excursion, the thickening of the diaphragm, and the speed of diaphragmatic contraction [10]. Among patients requiring mechanical ventilation, detection of diaphragmatic dysfunction by ultrasound performed during spontaneous breathing trial is associated with both longer weaning time and longer total ventilation time [11]. Ultrasound may be used to evaluate diaphragm excursions and also diaphragmatic thickness [9,12]. The evaluation of the diaphragmatic thickening fraction (DTF) may be helpful to assess diaphragmatic function and its contribution to respiratory workload [12]. However, to our knowledge, no studies have assessed diaphragmatic thickening as a predictor index for weaning from mechanical ventilation.

The aim of our study was to assess whether the degree of diaphragm thickening, measured by ultrasound during a weaning trial and expressed as thickening fraction, may be used to predict successful weaning.

## Methods

This was a prospective study, performed between December 2009 and December 2011 in the High Dependency Unit of the S. Giovanni Bosco Hospital in Turin, Italy. Patients were referred to our institution from the intensive care unit of our hospital after failing one or more attempts of weaning. The institutional ethic committee of the hospital approved the study. Patients gave their informed consent to participate in the study, which was conducted in accordance with the Declaration of Helsinki. Researchers involved in the study were all well trained in point-of-care ultrasound, with specific interest in lung ultrasound and diaphragm ultrasound imaging.

### Subjects

All patients were ventilated in pressure support ventilation through a tracheostomy tube. Patients underwent a spontaneous breathing trial when they met all the following criteria: FiO_2_ < 0.5, PEEP ≤5 cmH_2_O, PaO_2_/FiO_2_ > 200, respiratory rate <30 breaths per minute, absence of fever, alert and cooperative, and hemodynamic stability in the absence of any vaso-active therapy support. Subjects with diaphragm paralysis or neuromuscular diseases were excluded from the study.

### Study design

When the abovementioned criteria were reached, patients were disconnected from the tube and a spontaneous breathing trial was attempted for 1 h administering supplemental oxygen to achieve a peripheral oxygen saturation (SpO_2_) >94%. Then, each diaphragm was evaluated by B-mode and M-mode ultrasound subcostal views to rule out abnormalities in muscle movement [13]. When dysfunction of a single hemi-diaphragm was detected, patients were excluded from the study. Hereafter, right hemi-diaphragm ultrasound scans were performed with patients lying down at a semi-recumbent position (45°). Rapid shallow breathing index and maximum inspiratory pressure (PImax) were also recorded. Weaning failure was defined as the inability to maintain spontaneous breathing for at least 48 h, without any ventilatory support. Criteria for failure to the spontaneous breathing trial were the following: change in mental status, onset of discomfort, diaphoresis, respiratory rate (RR) >35 breaths/min, hemodynamic instability (heart rate >140, systolic blood pressure >180 or <90 mmHg), or signs of increased work of breathing [14]. Clinicians in charge of the patient's care were blinded to ultrasound measurements.

### Diaphragm ultrasound

All patients were evaluated in a semi-recumbent position. Ultrasound was performed using an Esaote MyLab 40 ultrasound system (Esaote, Genova, Italy) equipped with a 10-MHz linear probe.

Ultrasound evaluation was performed as previously described [15]. Briefly, the diaphragm was visualized by placing the transducer perpendicular to the chest wall, in the eighth or ninth intercostal space, between the anterior axillary and the midaxillary lines, to observe the zone of apposition of the muscle 0.5 to 2 cm below the costophrenic sinus. The diaphragm is imaged as a structure with three distinct layers, including two parallel echoic lines (the diaphragmatic pleura and the peritoneal membrane) and a hypoechoic structure between them (the muscle itself) [15,16]. The patient was then instructed to perform breathing to total lung capacity (TLC) and then to exhale to residual volume (RV). Several images of the diaphragm were captured and stored, including at least three at the point of maximum thickening at TLC and at least three at minimum thickening at RV.

On each frozen B-mode image, the diaphragm thickness was measured from the middle of the pleural line to the middle of the peritoneal line. Then, the DTF was calculated as percentage from the following formula: Thickness at end inspiration - Thickness at end expiration / Thickness at end expiration.

### Data analysis

Data were collected using Epi Info statistical software (Centers for Disease Control and Prevention, Atlanta, GA, USA) and analyzed using R version 2.15.0 (R Development Core Team: http://www.R-project.org).

Data are presented as mean (SD) or median [inter-quartile range] when appropriate. Descriptive statistics are shown for both the whole cohort and the subgroups of interest. Differences of continuous variables between the subgroups for the independent variable were assessed by non-parametric tests. The *χ*^2^ test, with Fisher correction when appropriate, was used for comparisons among categorical variables. Intra-observer variability was assessed measuring diaphragm thickness at least 24 h after the first measurement. To assess inter-observer variability, 23 patients were analyzed by two different operators. Pearson correlation analysis and Bland-Altman plotting were performed to evaluate the reproducibility of diaphragm ultrasound studies. Receiver operating characteristic (ROC) curve analysis was performed to assess diaphragm DTF ability to discriminate between patients who succeeded weaning and those who failed. The Spearman coefficient was used to evaluate correlations. A two-tailed *p* value of less than 0.05 was taken to indicate statistical significance.

## Results

During the study period, 75 patients were referred to the High Dependency Unit for weaning. Two showed signs of neuromuscular disorders and were excluded from the study. The remaining 73 were analyzed. Of these, 27 were weaned while the remaining 46 failed after the first spontaneous breathing trial. Table 1 summarizes the main clinical-demographic characteristics of the population enrolled in the study.

**Table 1:** Patient characteristics

	**All (*n*** **= 46)**	**Success (*n*** **= 29)**	**Failure (*n*** **= 17)**	***p *** **value**
Age	64.6 (12.1)	64.3 (13.7)	65.8 (10)	0.72
Male/female	34/12			
BMI	23 [22 to 27]	24 [22 to 27]	23 [20 to 26]	0.66
SAPS II	34 [33 to 39]	33 [29 to 45]	36 [33 to 43]	0.12
*D*_TLC_	0.34 [0.26 to 0.44]	0.38 [0.29 to 0.45]	0.30 [0.20 to 0.40]	0.08
*D*_RV_	0.24 [0.17 to 0.30]	0.25 [0.19 to 0.28]	0.24 [0.17 to 0.30]	0.81
DTF	0.38 [0.29 to 0.44]	0.56 [0.38 to 0.64]	0.26 [0.22 to 0.30]	<0.0001
RSBI	85 [65 to 112]	70 [57 to 83]	120 [110 to 148]	<0.0001
RR	28 [18 to 58]	27 [18 to 32]	31 [24 to 58]	0.001
PImax	67.5 (23.9)	82.9 (13.6)	41.2 (11.2)	<0.0001
Vte	340 [290 to 380]	360 [260 to 700]	280 [210 to 500]	0.007
HDU length of stay	15 [11 to 23]	15 [11 to 19]	22 [15 to 28]	0.02
Duration of ventilatory treatment	28 [22 to 37]	26 [19 to 30]	37 [28 to 45]	0.011
Mortality	3	1	2	

BMI, body mass index; SAPS, Simplified Acute Physiology Score; *D*_TLC_, diaphragm thickness at total lung capacity; *D*_RV_, diaphragm thickness at residual volume; DTF, diaphragm thickening fraction; RSBI, rapid shallow breathing index; RR, respiratory rate; PImax, maximum inspiratory pressure; Vte, expiratory tidal volume; HDU, high dependency unit.

Ultrasound examination was feasible in all patients, including those with body mass index >30. On occasion, the presence of pleural effusion or parenchymal lung consolidation did not affect the quality of ultrasound studies targeted to diaphragm evaluation.

**Figure 1:**
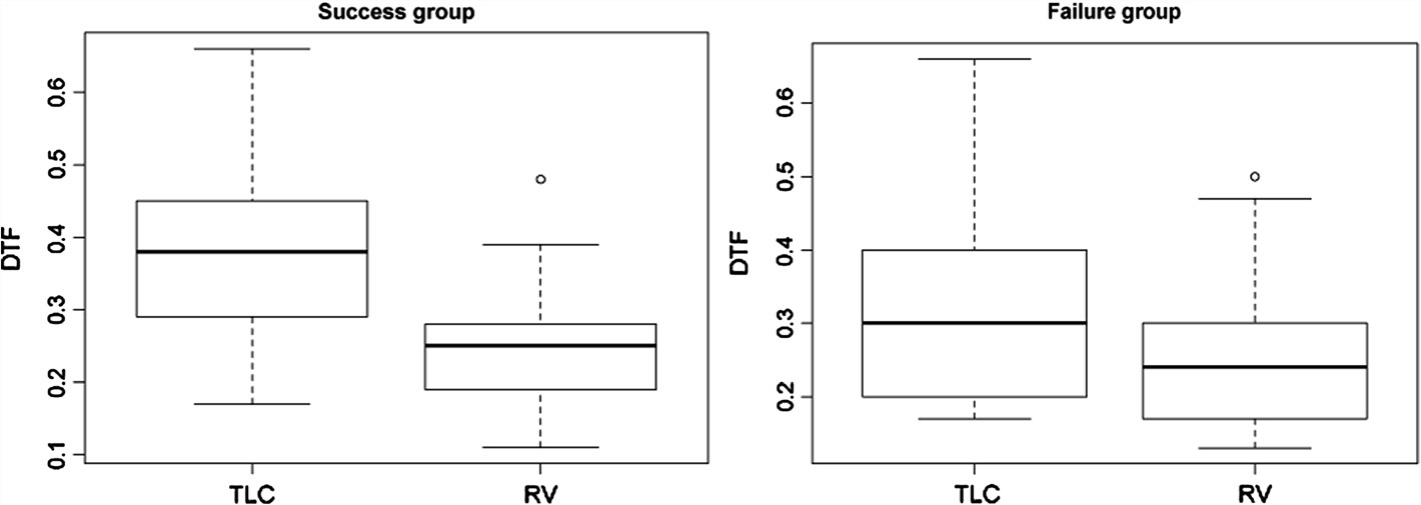
**Diaphragm thickness at TLC and RV in patients who failed and succeeded the spontaneous breathing trial.** Boxplot of diaphragm thickness: the central line represents the median value, the box boundaries represent the 25th and 75th percentiles, the whiskers represent the lowest datum within 1.5 inter-quartile range (IQR) of the lower quartile and the highest datum within 1.5 IQR of the upper quartile, and the circles represent outlier values. Diaphragm thickness at total lung capacity (TLC) and residual volume (RV) for patients who failed the spontaneous breathing trial: 0.30 [0.20 to 0.40] and 0.24 [0.17 to 0.30], *p* < 0.09. Diaphragm thickness at TLC and RV for patients who succeeded the spontaneous breathing trial: 0.38 [0.29 to 0.45] and 0.25 [0.19 to 0.28], *p* < 0.0001.

**Figure 2:**
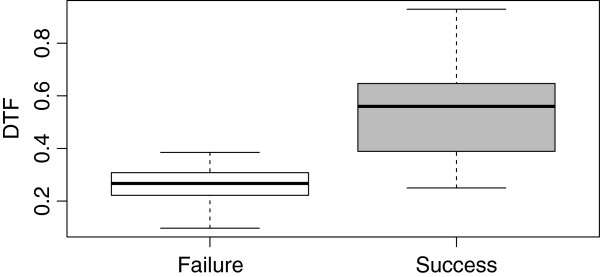
**Diaphragm thickening fraction (DTF) in patients who failed and succeeded the spontaneous breathing trial.** Boxplot of diaphragm thickening fraction: the central line represents the median value, the box boundaries represent the 25th and 75th percentiles, and the whiskers represent the lowest datum within 1.5 IQR of the lower quartile and the highest datum within 1.5 IQR of the upper quartile. DTF was 0.26 [0.22 to 0.30] and 0.56 [0.38 to 0.64], respectively (*p* < 0.0001).

**Figure 3:**
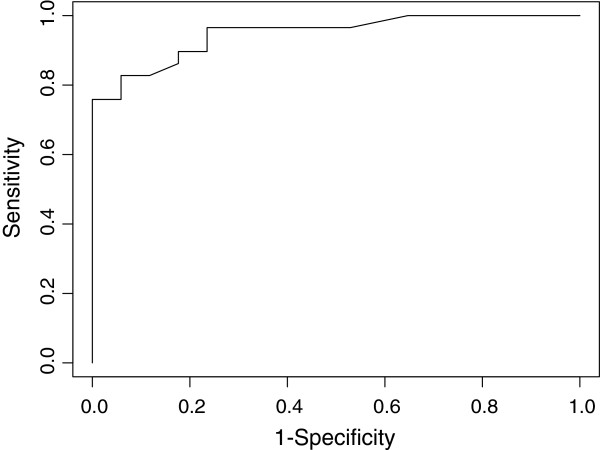
**Receiver operating characteristic curve for the diaphragm thickening fraction (DTF).** AUC 0.948 (95% CI 0.89 to 1.00).

Diaphragm thickness differed significantly between TLC and RV in patients who succeeded spontaneous breathing trial but not in patients who failed (Figure 1). Diaphragm thickness at both TLC and RV was positively correlated with body mass index (*rho* = 0.52, *p* = 0.0008 and *rho* = 0.57, *p* = 0.0002, respectively). A significant difference in DTF was observed between success and failure groups (Figure 2). A ROC curve was used to assess the diagnostic accuracy of DTF in predicting failure to the spontaneous breathing test (Figure 3). A cutoff value >36% was associated with a successful spontaneous breathing test with a sensitivity of 0.82, a specificity of 0.88, a positive predictive value (PPV) of 0.92, and a negative predictive value (NPV) of 0.75. By comparison, RSBI <105 showed a sensitivity of 0.93, a specificity of 0.88, a PPV of 0.93, and a NPV of 0.88 for determining success to the spontaneous breathing test.

**Figure 4:**
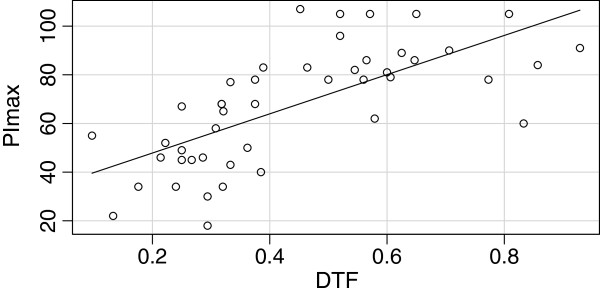
**Correlation between diaphragm thickening fraction (DTF) and maximum inspiratory pressure (PImax).** The circles represent measure of PImax and DTF for each single patient; rho = 0.75, *p* < 0.001.

A good correlation between DTF and PImax was observed (*rho* = 0.75, *p* < 0.001) (Figure 4).

Expiratory tidal volume was significantly different between success and failure groups, showing 360 ml [340 to 400 ml] vs 280 ml [236 to 300 ml], respectively, with *p* < 0.001. Moreover, DTF was positively correlated with expiratory tidal volume (*rho* = 0.55, *p* < 0.001). The duration of ventilatory treatment and hospital length of stay were significantly higher in patients who failed the spontaneous breathing test (Table 1).

### Intra- and inter-observer variability

Measurements of diaphragm thickness at both TLC and RV showed significantly high intra- and inter-observer correlations (*rho* = 0.98, *p* < 0.001 at TLC and *r* = 0.98, *p* < 0.001 at RV for intra-observer variability; *rho* = 0.96, *p* < 0.001 at TLC and *rho* = 0.98, *p* < 0.001 at RV for inter-observer variability).

## Discussion

To our knowledge, this is the first study that evaluates the DTF as a predictive index of weaning after a spontaneous breathing trial. The present study shows that a DTF >36% is associated with a successful weaning.

The important role of ultrasound in assessing diaphragm function has already been studied, but there are few data concerning the usefulness of diaphragm ultrasound evaluation as a weaning predictor.

In an elegant study, Lerolle and co-workers assessed the ultrasound criteria to determine diaphragmatic dysfunction after cardiac surgery [9]. They found that ultrasound determination of diaphragm excursion, expressed as the greater excursion of the muscle on a maximal inspiratory effort in patients requiring prolonged mechanical ventilation, may help identify those patients with severe diaphragmatic dysfunction. They also showed that their ultrasound measurement correlated well with trans-diaphragmatic pressure. However, this study was not designed to predict success of a weaning trial.

To assess the usefulness of diaphragmatic ultrasound in predicting successful weaning, Kim and co-workers studied the amplitude of diaphragm movements [11]. A diaphragm dysfunction was diagnosed by ultrasound if an excursion <10 mm or a paradoxical movement was observed. Diaphragmatic dysfunction was associated with weaning failure, and the authors conclude that ultrasound may identify patients at risk of difficult weaning.

Another ultrasound method, described since the last decade of the 1990s, is to evaluate the diaphragm thickness [15]. Ultrasound measures accurately the thickness of the muscle in the zone of apposition, with high reproducibility. Thickening ratio was reported to be a good indicator of diaphragm strength [15]. B-mode ultrasound may be used to assess the thickness of the muscle over a wide range of lung volumes from RV to TLC, as was demonstrated by Cohn and co-workers [16].

Diaphragm thickness may also be estimated in M-mode, although this method was criticized [16,17]. Nevertheless, Vivier and co-workers concluded that diaphragm thickness evaluated in M-mode is a non-invasive and reproducible ultrasound method, useful to evaluate muscle function and its contribution to respiratory workload [12]. However, the great majority of diaphragm ultrasound studies have measured diaphragm thickness in B-mode. Some evaluated the variation of thickness at different lung volumes from RV to TLC in normal subjects [15,18]. Another study measured diaphragm thickness in patients with diaphragm paralysis to monitor recovery of the muscle over time [19]. Interestingly, in this latter study, no thickening was observed by ultrasound in patients who did not recover from paralysis, thus providing useful information for both diagnosing diaphragm paralysis and indicating recovery.

We decided to measure diaphragm with B-mode ultrasound because this technique provides a greater anatomical definition of the muscle and its adjacent structures together with a more panoramic view in comparison with M-mode [16,18].

Conventional methods used to assess diaphragm function are the measurement of trans-diaphragmatic pressure (Pdi) and phrenic nerve stimulation. Also, fluoroscopy and electromyography have been largely used. However, all these methods are invasive and uncomfortable or expose the patients to radiations.

B-mode diaphragm ultrasound is a simple, rapid, reproducible, and non-invasive test that can be repeated several times without any risk for patients and provides important information on its respiratory function. Our data suggest that DTF has a potential in predicting those patients who may fail a weaning attempt, similarly to other already established weaning parameters and tests.

Our study has some limitations. The first is that, except with PImax, we did not perform a comparison with other methods that may be considered a gold standard in the assessment of diaphragmatic function, to validate ultrasound. A previous study found a good correlation between trans-diaphragmatic pressure-time product and DTF [12]. Although trans-diaphragmatic pressure-time may be considered a gold standard in studies that evaluate new tests of diaphragmatic function, it is highly invasive and uncomfortable for the patient. Furthermore, other studies already concluded that diaphragm ultrasound is a reliable method to evaluate its respiratory function, because measurements correlated well with lung volumes and with PImax [15,19]. Nevertheless, a future prospective trial comparing diaphragm ultrasound with trans-diaphragmatic pressure in patients with difficult weaning may be of high scientific interest and further validate this ultrasound method.

Another limitation of our study is the validity of the DTF cutoff that we obtained. We have studied a very selected population referred to our unit from the ICU of our hospital, after failure of a first gross attempt of weaning. This selection may only be obtained from the path specifically designed for our patients in our hospital. Our data cannot be automatically generalized to other populations of ordinary polyvalent ICUs. For this reason, other prospective studies should be designed to confirm the reliability of diaphragm US and to propose the most generalizable DTF threshold value.

## Conclusions

Ultrasound of the diaphragm is a simple method useful to evaluate the thickness of the muscle in the zone of apposition. This technique is highly feasible, harmless, and repeatable in the same patients. Assessment of DTF by diaphragm ultrasound in B-mode represents an easy-to-obtain new weaning index that, if further validated by other studies, may be introduced as a bedside method in the clinical practice.

## Competing interests

The authors declare that they have no competing interests.

## Authors’ contributions

GF conceived and designed the study; acquired, analyzed, and interpreted the data; drafted the manuscript and edited it for important intellectual and scientific content; served as the principal author; and edited the internal revision. GDF and FP acquired the data. FE acquired the data and edited the internal revision. FA conceived and designed the study and edited the internal revision. GV revised the article for important intellectual content. All authors read and approved the final manuscript.

## References

[B1] Esteban A Frutos F Tobin MJ Alia I Solsona JF Valverdù I Fernandez R De La Cal MA Benito S Tomàs R Carriedo D Macìas S Blanco J A comparison of four methods of weaning patients from mechanical ventilation. Spanish Lung Failure Collaborative Group N Engl J Med 1995 6 345 350 10.1056/NEJM199502093320601 7823995

[B2] El-Khatib MF Bou-Khalil P Clinical review: liberation from mechanical ventilation Crit Care 2008 6 221 10.1186/cc6959 18710593 PMC2575571

[B3] Yang KL Tobin MJ A prospective study of indexes predicting the outcome of trials of weaning from mechanical ventilation N Engl J Med 1991 6 1445 1450 10.1056/NEJM199105233242101 2023603

[B4] Epstein SK Etiology of extubation failure and the predictive value of the rapid shallow breathing index Am J Respir Crit Care Med 1995 6 545 549 10.1164/ajrccm.152.2.7633705 7633705

[B5] Lee KH Hui KP Chan TB Tan WC Lim TK Rapid shallow breathing (frequency-tidal volume ratio) did not predict extubation outcome Chest 1994 6 540 543 10.1378/chest.105.2.540 8306759

[B6] Krieger BP Isber J Breitenbucher A Throop G Ershowsky P Serial measurements of the rapid-shallow-breathing index as a predictor of weaning outcome in elderly medical patients Chest 1997 6 1029 1034 10.1378/chest.112.4.1029 9377913

[B7] Vassilakopoulos T Zakynthinos S Roussos C The tension-time index and the frequency/tidal volume ratio are the major pathophysiologic determinants of weaning failure and success Am J Respir Crit Care Med 1998 6 378 385 10.1164/ajrccm.158.2.9710084 9700110

[B8] Petrof BJ Jaber S Matecki S Ventilator-induced diaphragmatic dysfunction Curr Opin Crit Care 2010 6 19 25 10.1097/MCC.0b013e328334b166 19935062

[B9] Lerolle N Guerot E Dimassi S Zegdi R Faisy C Fagon JY Diehl JL Ultrasonographic diagnostic criterion for severe diaphragmatic dysfunction after cardiac surgery Chest 2009 6 401 407 10.1378/chest.08-1531 18753469

[B10] Matamis D Soilemezi E Tsagourias M Akoumianaki E Dimassi S Boroli F Richard JC Brochard L Sonographic evaluation of the diaphragm in critically ill patients. Technique and clinical applications Intensive Care Med 2013 6 801 810 10.1007/s00134-013-2823-1 23344830

[B11] Kim WY Suh HJ Hong SB Koh Y Lim CM Diaphragm dysfunction assessed by ultrasonography: influence on weaning from mechanical ventilation Crit Care Med 2011 6 2627 2630 21705883 10.1097/CCM.0b013e3182266408

[B12] Vivier E Mekontso Dessap A Dimassi S Vargas F Lyazidi A Thille AW Brochard L Diaphragm ultrasonography to estimate the work of breathing during non-invasive ventilation Intensive Care Med 2012 6 796 803 10.1007/s00134-012-2547-7 22476448

[B13] Boussuges A Gole Y Blanc P Diaphragmatic motion studied by m-mode ultrasonography: methods, reproducibility, and normal values Chest 2009 6 391 400 10.1378/chest.08-1541 19017880

[B14] MacIntyre NR Cook DJ Ely EW Jr Epstein SK Fink JB Heffner JE Hess D Hubmayer RD Scheinhorn DJ Evidence-based guidelines for weaning and discontinuing ventilatory support: a collective task force facilitated by the American College of Chest Physicians; the American Association for Respiratory Care; and the American College of Critical Care Medicine Chest 2001 6 375S 395S 10.1378/chest.120.6_suppl.375S 11742959

[B15] Ueki J de Bruin PF Pride NB In vivo assessment of diaphragm contraction by ultrasound in normal subjects Thorax 1995 6 1157 1161 10.1136/thx.50.11.1157 8553271 PMC475087

[B16] Cohn D Benditt JO Eveloff S McCool FD Diaphragm thickening during inspiration J Appl Physiol 1997 6 291 296 9216975 10.1152/jappl.1997.83.1.291

[B17] Wait JL Johnson RL Patterns of shortening and thickening of the human diaphragm J Appl Physiol 1997 6 1123 1132 9338420 10.1152/jappl.1997.83.4.1123

[B18] Boon AJ Harper CJ Ghahfarokhi LS Strommen JA Watson JC Sorenson EJ Two-dimensional ultrasound imaging of the diaphragm: quantitative values in normal subjects Muscle Nerve 2013 6 884 889 10.1002/mus.23702 23625789

[B19] Summerhill EM El-Sameed YA Glidden TJ McCool FD Monitoring recovery from diaphragm paralysis with ultrasound Chest 2008 6 737 743 10.1378/chest.07-2200 18198248

